# Exploring physicochemical characteristics of cyclodextrin through M-polynomial indices

**DOI:** 10.1038/s41598-024-68775-z

**Published:** 2024-08-28

**Authors:** Abdul Rauf, Muhammad Naeem, Rahila Ramzan, Alhagie Cham

**Affiliations:** 1 Department of Mathematics, Air University Multan Campus, Multan, Pakistan; 2 Department of Mathematics, School of Natural Sciences, National University of Sciences and Technology (NUST), Islamabad, Pakistan; 3 School of Arts and Sciences, University of The Gambia, Banjul, The Gambia

**Keywords:** Chemical graph theory, Physic-chemical characteristics, Degree-based M-polynomial indices, Cyclodextrin, Biochemistry, Diseases, Mathematics and computing

## Abstract

Cyclodextrin, a potent anti-tumor medication utilized predominantly in ovarian and breast cancer treatments, encounters significant challenges such as poor solubility, potential side effects, and resistance from tumor cells. Combining cyclodextrin with biocompatible substrates offers a promising strategy to address these obstacles. Understanding the atomic structure and physicochemical properties of cyclodextrin and its derivatives is essential for enhancing drug solubility, modification, targeted delivery, and controlled release. In this study, we investigate the topological indices of cyclodextrin using algebraic polynomials, specifically the degree-based M-polynomial and neighbor degree-based M-polynomial. By computing degree-based and neighbor degree-based topological indices, we aim to elucidate the structural characteristics of cyclodextrin and provide insights into its physicochemical behavior. The computed indices serve as predictive tools for assessing the health benefits and therapeutic efficacy of cyclodextrin-based formulations. In addition, we examined that the computed indices showed a significant relationship with the physicochemical characteristics of antiviral drugs. Graphical representations of the computed results further facilitate the visualization and interpretation of cyclodextrin's molecular structure, aiding researchers in designing novel drug delivery systems with improved pharmacological properties.

## Introduction

One of the most important areas of current medical study is the effective treatment of cancer. The main surgical technique used presently for determining the cause and cure of cancer is chemotherapy. Due to its widespread adoption, efficacy and frequent application in the treatment of cancer, chemotherapy has gained enormous appeal. The physiological mechanisms of medication action, covering respiration, transport, acceptance and urine, are generally well understood in contemporary health research, leading to sophisticated clinical applications^1,2^. Chemotherapy, a widely used treatment for cancer, is hindered by its severe side effects and the potential to propagate infectious diseases. These limitations impose restrictions on the efficacy of numerous medications currently available. Despite the pressing demand for solutions, there is a lack of appropriate formulations or clinical models to effectively address these challenges in the realm of successful cancer treatment^3–5^. Some experimental chemotherapeutic drugs have also had difficulty being effectively converted into therapeutic uses due to challenges in achieving tumor selectivity and preventing systemic toxicity. To decrease irregular reactions to cancer treatment, active targeting of tumor cells is essential. Previous research has outlined distinct structural, pathophysiological and micro-environmental differences that can be introduced between malignant regions and healthy body tissues to achieve this^6,7^. The characteristics of drugs or chemicals can be examined by their structure topology.

In the branch of mathematical chemistry, referred to as "chemical graph theory", chemical interactions are studied quantitatively using methodologies from graph theory. It involves the application of graph theory to address complex molecular challenges, weaving together nontrivial graph theory applications^8,9^. The theory concerning chemical graphs is widely applied in the realm of chemical science. It is important when researching the quantitative structure–property relationship (QSPR) and the quantitative structure–activity relationship (QSAR). Researchers focused on understanding and predicting the physico-chemical characteristics as well as the biological activity of numerous types of pharmaceuticals^10^.

Chemical graph theory states that the edges of the molecular graph are bonds of chemicals and atoms are its nodes/vertices. These representations synthesize a chemical network into a specific number. Because of their intimate relationship to chemicals, topological indices are frequently utilized in chemical graph theory. One of the fundamental topological indices is based on vertex degrees, which is the sum of the number of edges that connect to each vertex. When defining and outlining the atomic structure of chemical substances, this measure is often employed. Scholars commonly use topological indices to better understand and clarify the statistical attributes of medications^11–14^. The topological indices, which provide comprehensive details about the interaction and order of elements within molecules, enable better comprehension of the thermodynamic and physiological properties of substances.

In the studies on paraffin, Wiener was the first researcher to propose the Wiener index^11^. Setting the groundwork for their future use in the explanation and analysis of chemical structures, this was a significant development in the study of topological indices.

Milan Randić, who was born in 1975, proposed the Randić index^12^. The generalized Randić index was developed in 1998 by Erdos, Ballobás, and Amic^15,16^. Among these, the Randić index is the most popular and extensively researched topological index and it is widely used in many scientific studies.

The Harmonic index, developed initially by Zhong^17^ is a variation of the Randić index. Ediz et al.^18^ proceeded to present a modified Harmonic index. Gutman and Trinajstic invented the first and second Zagreb index, and later they defined the modified second Zagreb index^19–22^. In the realm of chemical & mathematical literary works, the topological indices most commonly used are the Randić, Zagreb, and Wiener indices^16,23,24^. To complement these, several additional degree-based topological indices have been developed, including the augmented Zagreb, reverse sum-indeg, and symmetric division index^25–28^. Significantly, in 2015, Furtula and Gutman presented another topological index named "F-index" or "forgotten index"^25^.

A full comprehension of the structure of molecules and their mathematical features is aided by this wide range of topological indices. Algebraic polynomials hold significant importance in the field of chemistry. The Hosoya polynomial, first presented by H. Hosoya in 1988^26^, is one example of such a polynomial. Many physicochemical properties related to organic compounds can be estimated using degree-based and distance-based graph invariants. In chemical graph theory, many polynomials have been introduced like, PI polynomials^27^, Clar covering polynomials^28^, Schultz polynomials^29^, Tutte Polynomials^30^, theta polynomials^31^ and more.

Among these, the ${\mathcal{M}}$-polynomial, introduced in 2015 by Deutsch and Klavzar^32^, plays a crucial role in computing degree-based topological indices. It acts identically to the production of distance-based indices in the aforementioned scenario, as observed with Hosoya polynomials. Subsequent studies on ${\mathcal{M}}$-polynomials have further explored their applications. Munir et al. computed degree-based topological indices and ${\mathcal{M}}$-polynomials for a variety of frameworks, including dendrimers, small stars, upright and weave polynomials nanotubes, and single-walled carbon nanotubes and various groups of revolving networks. Applying ${\mathcal{M}}$-polynomials, Geo et al. elaborated on this work to create topological measurements for the crystalline forms of iron difluoride as well as copper oxide^33–36^. Many researchers examined that degree-based ${\mathcal{M}}$-polynomials indices show significant results in the analysis of diverse molecular structures. Table 1 presents a comprehensive description of the formulation procedure of degree-based topological indices employing the ${\mathcal{M}}$-polynomial formulation.

**Table 1:** The mathematical formulas of ${\mathcal{M}}$-polynomial degree-based indices.

Index name	Degree based formula	Formulation
First Zagreb index (M_1_)	$\sum_{\text{rs} \epsilon E}{\rho}_{r}+{\rho}_{s}$	( ${\Delta }_{\text{c}}$+${\Delta }_{\text{d}}$)(${\mathcal{M}}$(G))|_c, d=1_
Second Zagreb index (M_2_)	$\sum_{\text{rs} \epsilon E}{\rho}_{r}{\rho}_{s}$	(${\Delta }_{\text{c}}{\Delta }_{\text{d}}$)(${\mathcal{M}}$(G))|_c, d=1_
Modified second Zagreb index (^m^M_2_)	$\sum_{\text{rs} \epsilon E}\frac{1}{{\rho}_{r}{\rho}_{s}}$	(${\text{I}}_{\text{c}}$ ${\text{I}}_{\text{d}}$ ) (${\mathcal{M}}$(G))|_c, d=1_
Augmented Zagreb index (AZI)	$\sum_{\text{rs} \epsilon E}({\frac{{\rho}_{r}{\rho}_{s}}{{\rho}_{r}+{\rho}_{s}-2})}^{3}$	${{\varvec{I}}}_{{\varvec{c}}}^{3}$ Q_-2_ J ${\Delta }_{{\varvec{c}}}^{3}{\Delta }_{{\varvec{d}}}^{3}$ (${\mathcal{M}}$(G))|_c=1_
General Randić index (GR_α_)	$\sum_{\text{rs} \epsilon E}{\rho}_{r}^{\alpha }+{\rho}_{s}^{\alpha }$	${\Delta }_{{\varvec{c}}}^{\boldsymbol{\alpha }}{\Delta }_{{\varvec{d}}}^{\boldsymbol{\alpha }}$a. (${\mathcal{M}}$(G))|_c, d=1_
Harmonic index (H)	$\sum_{\text{rs} \epsilon E}\frac{2}{{\rho}_{r}+{\rho}_{s}}$	2I_c_J(${\mathcal{M}}$ (G))|_c=1_
Inverse sum indeg index (ISI)	$\sum_{\text{rs} \epsilon E}\frac{{\rho}_{r}{\rho}_{s}}{{\rho}_{r}+{\rho}_{s}}$	${\text{I}}_{\text{c}}$ J ${\Delta }_{\text{c}}{\Delta }_{\text{d}}$ (${\mathcal{M}}$(G))|_c=1_
Symmetric division deg index (SSD)	$\sum_{\text{rs} \epsilon E}\frac{{\rho}_{r+}^{2}{\rho}_{s}^{2}}{{\rho}_{r}+{\rho}_{s}}$	(${\Delta }_{\rm{c}}{\text{I}}_{\rm{d}}$ + ${{\text{I}}_{\text{c}}\Delta }_{\text{d}}$)(${\mathcal{M}}$(G))|_c, d=1_
Forgotten topological index (F)	$\sum_{\text{rs} \epsilon E}{\rho}_{r+}^{2}{\rho}_{s}^{2}$	(${\Delta }_{{\varvec{c}}}^{2\boldsymbol{ }}{+\Delta }_{{\varvec{d}}}^{2}$)(${\mathcal{M}}$(G))|_c, d=1_
Redefine third Zagreb index (ReZG_3_)	$\sum_{\text{rs} \epsilon E}{\rho}_{r}{\rho}_{s}({\rho}_{r}+{\rho}_{s})$	${\Delta }_{\text{c}}{\Delta }_{\text{d}}$(${\Delta }_{\text{c}}$+${\Delta }_{\text{d}}$)(${\mathcal{M}}$(G))|_c, d=1_

In their research, Monda et al.^34^ introduced the concept of the neighborhood ${\mathcal{M}}$-polynomial, a tool designed for computing neighborhood degree sum-based topological indices. These indices serve a purpose analogous to that of the degree-based ${\mathcal{M}}$-polynomial. The application of neighborhood polynomials extends to the examination of topological indices for structures such as bismuth tri-iodide sheets and chains, where the focus is on neighborhood-degree sum-based analyses.

The utility of neighborhood degree sum-based topological indicators goes beyond conventional degree-based approaches, offering enhanced precision in predicting various physical, chemical, and medicinal properties^12,37,38^. Table 2 provides a comprehensive overview of the topological indices derived from the neighborhood ${\mathcal{M}}$-polynomial, specifically those based on neighborhood degree sums. Table 3 shows the division of P according to the degree of each edge.

**Table 2:** The neighborhood degree-based M-polynomial ($${\mathcal{N}}$$$${\mathcal{M}}$$-polynomials) indices.

Index name	Nbhd degree based formula	Formulation
Nbhd first Zagreb index (^n^${\mathcal{M}}$_1_)	$\sum_{\text{rs} \epsilon E}{\mathcal{N}}(\text{r})+{\mathcal{N}}(\text{s})$	(${\Delta }_{\text{c}}$+${\Delta }_{\text{d}}$)(${\mathcal{N}}\, {\mathcal{M}}$(G))|_c, d=1_
Nbhd second Zagreb index (^n^${\mathcal{M}}$_2_)	$\sum_{\text{rs} \epsilon E}{\mathcal{N}}(\text{r}){\mathcal{N}}(\text{s})$	(${\Delta }_{\text{c}}{\Delta }_{\text{d}}$)(${\mathcal{N}} {\mathcal{M}}$ (G))|_c, d=1_
Nbhd modified second Zagreb index (^nm^${\mathcal{M}}$_2_)	$\sum_{\text{rs} \epsilon E}\frac{1}{{\mathcal{N}}(\text{r}){\mathcal{N}}(\text{s})}$	(${\text{I}}_{\rm{c}}{\text{I}}_{\rm{d}}$) (${\mathcal{N}} {\mathcal{M}}$ (G))|_c, d=1_
Nbhd augmented Zagreb index (^n^AZI)	$\sum_{\text{rs} \epsilon E}({\frac{{\mathcal{N}}(\text{r}){\mathcal{N}}(\text{s})}{{\mathcal{N}}(\text{r}) +{\mathcal{N}}(\text{s})-2})}^{3}$	${I}_{c}^{3}$ Q_−2_ J ${\Delta }_{{\varvec{c}}}^{3}{\Delta }_{{\varvec{d}}}^{3}$ (${\mathcal{N}} {\mathcal{M}}$ (G))|_c=1_
Nbhd general Randić index (^n^GR_α_)	$\sum_{\text{rs} \epsilon E}{\mathcal{N}}{(r)}^{\alpha }{\mathcal{N}}{(s)}^{\alpha }$	${\Delta }_{{\varvec{c}}}^{\boldsymbol{\alpha }}{\Delta }_{{\varvec{d}}}^{\boldsymbol{\alpha }}$( ${\mathcal{N}} {\mathcal{M}}$ (G))|_c, d=1_
Nbhd harmonic index (^n^H)	$\sum_{\text{rs} \epsilon E}\frac{2}{{\mathcal{N}}\left(\text{r}\right)+{\mathcal{N}}(\text{s})}$	2I_c_J( ${\mathcal{N}} {\mathcal{M}}$ (G))|_c=1_
Nbhd inverse sum indeg index (^n^ISI)	$\sum_{\text{rs} \epsilon E}\frac{{\mathcal{N}}(\text{r}){\mathcal{N}}(\text{s})}{{\mathcal{N}}(\text{r}) +{\mathcal{N}}(\text{s})}$	${\text{I}}_{\text{c}}$ J ${\Delta }_{\text{c}}{\Delta }_{\text{d}}$ (${\mathcal{N}} {\mathcal{M}}$(G))|_c=1_
Nbhd symmetric division deg index (^n^SSD)	$\sum_{\text{rs} \epsilon E}\frac{{\mathcal{N}}{(r)}^{2}+{\mathcal{N}}{(s)}^{2}}{{\mathcal{N}}(\text{r}){\mathcal{N}}(\text{s})}$	(${\Delta }_{\rm{c}}{\text{I}}_{\rm{d}}$+I_c_ ${\Delta }_{\text{d}}$)(${\mathcal{N}} {\mathcal{M}}$ (G))|_c, d=1_
Nbhd forgotten topological index (^n^F)	$\sum_{\text{rs} \epsilon E}{\mathcal{N}}{(r)}^{2}+{\mathcal{N}}{(s)}^{2}$	(${\Delta }_{{\varvec{c}}}^{2}{\Delta }_{{\varvec{d}}}^{2}$)(${\mathcal{N}} {\mathcal{M}}$ (G))|_c, d=1_
Nbhd redefine third Zagreb index (^n^ReZG_3_)	$\sum_{\text{rs} \epsilon E}{\mathcal{N}}(\text{r}){\mathcal{N}}(\text{s})({\mathcal{N}}\left(\text{r}\right)+{\mathcal{N}}\left(\text{s}\right))$	${\Delta }_{\text{c}}{\Delta }_{\text{d}}$(${\Delta }_{\text{c}}$+${\Delta }_{\text{d}}$)(${\mathcal{N}} {\mathcal{M}}$(G))|_c, d=1_

**Table 3:** Partition of P according to the degree of end vertices of each edge.

U_(a ,b)_	ρ_a,_ ρ_b_	Frequency
U_(1,2)_	1,2	3n + 15
U_(2,4)_	2,4	7n + 35
U_(1,4)_	1,4	7n + 35
U_(4,4)_	4,4	5n + 25

The study aims to investigate several topological indices of the cyclodextrin-conjugated molecular structure of a medicinal compound used as an anticancer drug. This research contributes to a deeper comprehension of the structural and functional characteristics of the examined molecular entity. After deriving $${\mathcal{M}}$$-polynomials & $${\mathcal{N}}$$$${\mathcal{M}}$$-polynomials for a specific structure, we analyzed the degree-based & neighborhood degree sums.

### Preliminaries

In graph theory, let L = (V,E) represent a graph where E is the set of edges and V is the set of vertices. A vertex, denoted as s, is an element of V, and an edge, denoted as rs = e, is an unordered pair distinct from V. The degree of vertex s, denoted as ρ_s,_ is the number of edges incident to v. The open neighborhood degree of a vertex, denoted as ${\mathcal{N}}\left(s\right)$, is the cardinality of the set of vertices adjacent to s (excluding s itself). The closed neighborhood degree of a vertex ‘s’ denoted as ${\mathcal{N}}\left[s\right]$, is the cardinality of the set of vertices adjacent to ‘s’ (including s) or the sum of ρ_s_ and ${\mathcal{N}}\left(s\right)$.

A graph L of $${\mathcal{M}}$$-polynomials represented by $${\mathcal{M}}$$$$\left(L\right)$$, of any edges rs ϵ E (G) where (degree of vertex r) ρ_r_ = a, (degree of vertex s) ρ_s_ = b, describes as:

$${\mathcal{M}}\left(L\right)=\sum_{a\le b}|{{\mathcal{N}}}_{(a, b)}|{c}^{a}{d}^{b}$$

where |${\mathcal{N}}$_(a, b)_| is the sum of the quantity of (a, b).

The neighborhood (Nbhd) degree sum of r vertex, represented by ${\mathcal{N}}\left(r\right)$, is the sum of the degree of all open Nbhd of r, e.g.

$${\mathcal{N}}\left(r\right)=\sum_{s\epsilon {\mathcal{N}}(r)}{\rho}_{s}$$

The Nbhd $${\mathcal{M}}$$-polynomial graph L designated by $${\mathcal{N}}$$$${\mathcal{M}}$$ (G) some edges rs $${\mathcal{M}}$$ $$L\left(E\right)$$ where $${\mathcal{N}}$$(r) = g,

$${\mathcal{N}}$$(s) = h, defined as

$${\mathcal{N}}{\mathcal{M}}(L)=\sum_{g\le h}|{{{\mathcal{N}}}^{p}}_{(g, h)}|{c}^{g}{d}^{h}$$

where |${{{\mathcal{N}}}^{p}}_{(g, h)}$| is the sum of (g,h) edges.

Numerous graph theory procedures, such as polynomials, graph invariants, and eigenvalues, are crucial in many different kinds of contexts.

Where $${\Delta }_{\text{c}}$$ = c $$\frac{\partial P(c,d)}{\partial c}$$, $${\Delta }_{\text{d}}$$= d $$\frac{\partial P(c,d)}{\partial d}$$, I_x_ = $$\underset{0}{\overset{c}{\int }}\frac{P(t,d)}{t}dt, {\text{ I}}_{\text{y}}$$ $$=\underset{0}{\overset{d}{\int }}\frac{P(t,d)}{t}dt$$, J $$\left(P(c,d)\right)$$ =$$P\left(c,c\right),$$ Q_α_ (P(c, d)) = c^2^P(c, d) gives the representation surface of $${\mathcal{M}}$$-polynomial and $${\mathcal{N}}$$$${\mathcal{M}}$$-polynomial molecular structure.

## Methodology

In this study, molecular graphs were employed as models for representing the structure of cyclodextrins conjugate. The molecular structures, particularly the conjugate structures of cyclodextrins, were computed using algebraic polynomials. The computations involved both degree-based and neighborhood degree sum-based values, achieved through various mathematical operations. Two types of polynomials, namely $${\mathcal{M}}$$ and $${\mathcal{N}}$$$${\mathcal{M}}$$, were utilized in the calculations. The methodology employed a combinatorial processing strategy, edge partition technique, vertex partition technique, and techniques for counting degrees. Additionally, entire approaches for assessing degree neighbors were applied. The computational tasks were verified using MATLAB programming, incorporating numerical calculations. To enhance the visual representation of the results Maple was employed. It facilitated the surface plotting of $${\mathcal{M}}$$-polynomials and $${\mathcal{N}}$$$${\mathcal{M}}$$-polynomials, as well as two-dimensional plotting. These graphical representations provided a clear visualization of the degree-based and neighbor degree sum-based numerical results, contributing to a comprehensive analysis of the molecular structures under investigation. For the QSPR, we used SPSS for linear regression models.

In short, we outline our key computations. We calculate the $${\mathcal{M}}$$ and $${\mathcal{N}}$$$${\mathcal{M}}$$-polynomials of the conjugated Cyclodextrin (CD) molecular structure. Key results using the pharmaceutical polymer conjugate have been discussed, including topological indices of certain anti-cancer drug models.

## Cyclodextrin

One type of cyclic oligosaccharide known as cyclodextrin is unique in that it is composed of several glucose molecules joined together to produce a distinctive cone-shaped cylindrical structure. This form has a hydrophobic central chamber and a series of hydrophilic outside rings^37^. The presence of lipophilic functional groups in the inner cavity is remarkable, as it greatly improves the ability to dissolve, permeability, and stability of the molecule. This change further decreases fluctuations, which in turn decreases the odour^37^. Cyclodextrin is a well-known and adaptable substance that enhances the ability of a medicine to dissolve in water and its stability while shielding the drug's chemical makeup and intended physiological occupation^38^. Furthermore, interacting with harmful chemicals like phosphobenzene, bisphenol-A, biphenyl, and other dangerous compounds is helpful when using them^39^. The unique qualities of cyclodextrin allow it to be used in ecological engineering projects by quickly cleaning sewage and decreasing pollution levels in the surroundings^40^.

**Figure 1:**
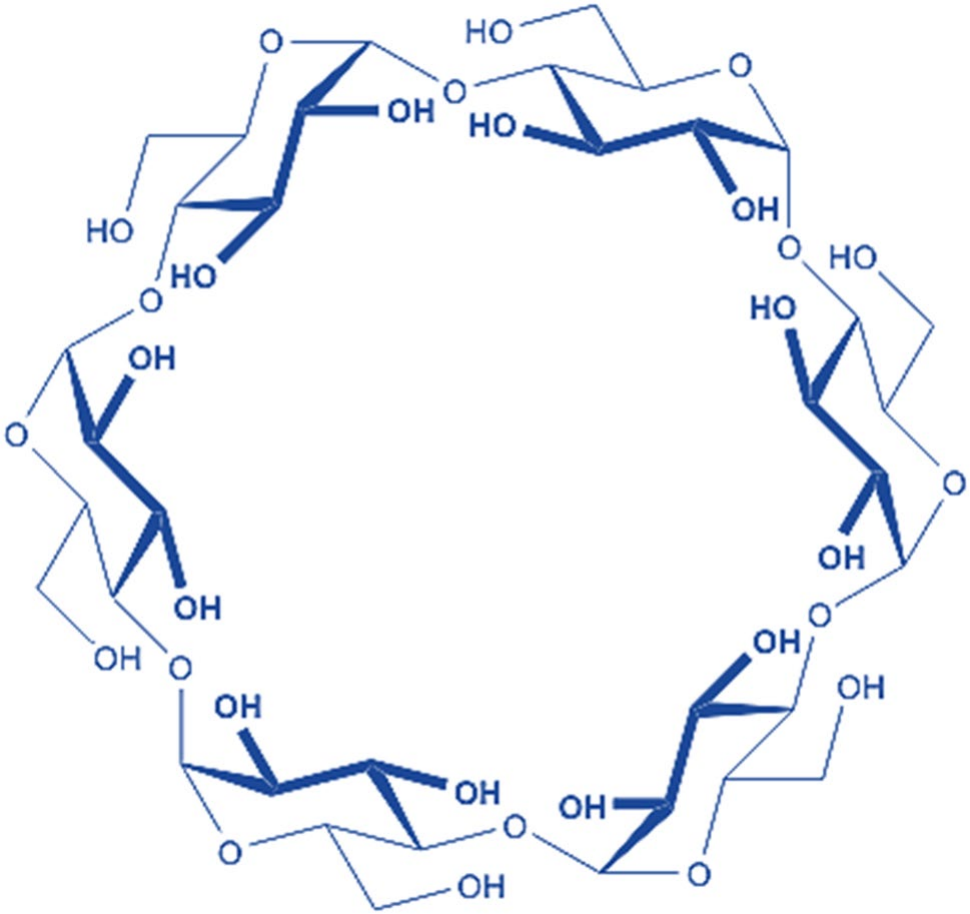
Molecular structure of the Cyclodextrin.

Applications for cyclodextrin can be found in an extensive variety of industries, including medical products, food, beauty products, and echological engineering. In conclusion, cyclodextrins' many advantages underscore their vitality and widespread impact on boosting solutions across a range of fields. Figure 1 shows the molecular structure of the Cyclodextrin.

### Theorem 1

*Let P be a molecular graph representing the conjugate structure of cyclodextrins. The polynomial equation of P is*

$${\mathcal{M}} (\text{P};{\text{c,d}}) = (3\text{cd}^{2}+7\text{c}^{2}\text{d}^{4}+7\text{cd}^{4}+5\text{c}^{4}\text{d}^{4})\text{ n}+15\text{cd}^{2}+35\text{c}^{2}\text{d}^{4} +35\text{c}^{1}\text{d}^{4}+25\text{c}^{4}\text{d}^{4}.$$

### ***Proof***

Let P represent the chemical configuration of cyclodextrin conjugates. The P have |E (P)|= 22n + 110 edges and |V (P)|= 21n + 105 vertices, with vertices of degrees V_1_, V_2_, V_3_, and V_4_, respectively. Where |V_1_|= 10n + 50, |V_2_|= 5n + 25, |V_3_|= 0.n + 0, and |V_4_|= 6n + 30. Consider the set

${\mathcal{N}}$(_a, b)_ = {$rs$ ϵE (P) | ρ_r_ = a ρ_s_ = b}, which is composed of all edges with end degree of vertices(a, b). We separate edges into four groups based on degrees, such as

$${\mathcal{N}}_{(1, 2)} = \{{\text rs} \epsilon {\rm E} |{\rho}\text{r }= 1,{\rho}\text{s }=2\},{\mathcal{N}}_{(2, 4)} = \{{\text rs} \epsilon {\rm E} |{\rho}\text{r }= 2,{\rho}\text{s }=4\},$$

$${\mathcal{N}}_{(1, 4)} = \{{\text rs} \epsilon {\rm E} |{\rho}\text{r }= 1,{\rho}\text{s }=4\},{\mathcal{N}}_{(4, 4)} = \{{\text rs} \epsilon {\rm E} |{\rho}\text{r }= 4,{\rho}\text{s }=4\}$$

Afterward, we obtain

$$\left|{\mathcal{N}}_{\left(1, 2\right)}\right|= 3\text{n}+15, \left|{\mathcal{N}}_{\left(2, 4\right)}\right|=7\text{n}+35, \left|{\mathcal{N}}_{\left(1, 4\right)}\right|=7\text{n}+35, \left|{\mathcal{N}}_{\left(4, 4\right)}\right|=5\text{n}+25.$$

The ${\mathcal{M}}$ polynomial, by definition, provides us with:

$${\mathcal{M}} (\text{P}) = \sum_{a\le b}|{{\mathcal{N}} }_{\left(a, b\right)}|{c}^{a}{d}^{b}=|{\mathcal{N}}(1, 2) |\text{ c}^{1}\text{d}^{2} + |{\mathcal{N}}(2, 4) |\text{ c}^{2}\text{d}^{4} + |{\mathcal{N}}(4, 1) |\text{ c}^{1}\text{d}^{4} + |{\mathcal{N}}(4, 4) |\text{ c}^{4}\text{d}^{4}$$

$${\mathcal{M}} (\text{P})= (3\text{n}+15)\text{c}^{1}\text{d}^{2}+ (7\text{n}+35)\text{c}^{2}\text{d}^{4}+ (7\text{n}+35)\text{c}^{1}\text{d}^{4} + (5\text{n}+25)\text{c}^{4}\text{d}^{4}$$

$${\mathcal{M}} (\text{P};\text{ c},\text{ d}) = (3\text{cd}^{2}+7\text{c}^{2}\text{d}^{4}+7\text{ c}^{4}\text{d}^{1}+5\text{c}^{4}\text{d}^{4})\text{ n}+15\text{cd}^{2}+35\text{c}^{2}\text{d}^{4} +35\text{c}^{1}\text{d}^{4}+25\text{c}^{4}\text{d}^{4}$$

$$\square$$

### Theorem 2

*Let P be a molecular graph of cyclodextrin conjugate. Then*

- *First Zagreb index* (M_1_) = 126n + 630,
- *Second Zagreb index* (M_2_) = 170n + 830,
- *Forgotten topological index* (F) = 434n + 2170,
- *Redefine third Zagreb index* (ReZG_3_) = 1134n + 5670,
- *General Randić Index* (GR_α_) = 2^α^ (3n + 15) + 8^α^ (7n + 35) + 4 ^α^ (7n + 35) + 16 ^α^ (5n + 25),
- *Modified second Zagreb index* (^m^M_2_) = $\frac{61}{16}$ n + $\frac{355}{16}$,
- *Symmetric division deg index* (SDD) = $\frac{259}{4}$ n + $\frac{1295}{4}$,
- *Harmonic index* (H) = $$\frac{503}{60}$$ n + $$\frac{483}{12}$$,
- *Inverse sum indeg index* (ISI) = $\frac{404}{15}$ n + $\frac{404}{3}$,
- *The augmented Zagreb index* (AZI) = ($\frac{5168}{27}$) n + $\frac{25840}{27}$.

### ***Proof***

By Using Theorem 1 polynomial and formula written in Table 1, we can easily prove theorem. Let the polynomial

$$\text{F}\left({\text{c,d}}\right)= \left(3\text{n}+15\right)\text{c}^{1}\text{d}^{2}+ \left(7\text{n}+35\right)\text{c}^{2}\text{d}^{4} + \left(7\text{n}+35\right)\text{c}^{1}\text{d}^{4} + \left(5\text{n}+25\right)\text{c}^{4}\text{d}^{4}$$

Then,

- First Zagreb index (M_1_)
  $$({\Delta }_{\text{c}}+{\Delta }_{\text{d}})({\mathcal{M}} (\text{G})) |_{\text{c},\text{ d}=1}=\text{ M}_{1}$$
  $$\left({\Delta }_{\text{c}}+{\Delta }_{\text{d}}\right)\left(\text{F}\left({\text{c,d}}\right)\right)= 3\left(3\text{n}+15\right)\text{c}^{1}\text{d}^{2}+6\left(7\text{n}+35\right)\text{c}^{2}\text{d}^{4}+ 5\left(7\text{n}+35\right)\text{c}^{1}\text{d}^{4} + 8 \left(5\text{n}+25\right)\text{c}^{4}\text{d}$$
  $$({\Delta }_{\text{c}}+{\Delta }_{\text{d}})(\text{F}({\text{c,d}})) |_{\text{c},\text{ d}=1}=9\text{n }+45+42\text{n}+210+35\text{n}+175+40\text{n}+200+126\text{n}+630$$
  $$\text{M}_{1}=126\text{n}+630.$$
- Second Zagreb index (M_2_)
  $$({\Delta }_{\text{c}}{\Delta }_{\text{d}}) ({\mathcal{M}} (\text{G})) |_{\text{c},\text{ d}=1} =\text{ M}_{2}$$
  $$({\Delta }_{\text{c}}{\Delta }_{\text{d}}) (\text{F}\left({\text{c,d}}\right)= 2\left(3\text{n}+15\right)\text{c}^{1}\text{d}^{2}+8\left(7\text{n}+35\right)\text{c}^{2}\text{d}^{4}+4 \left(7\text{n}+35\right)\text{c}^{1}\text{d}^{4} + 16\left(5\text{n}+25\right)\text{c}^{4}\text{d}^{4}$$
  $$\left({\Delta }_{\text{c}}{\Delta }_{\text{d}}\right)\left(\text{F}\left({\text{c,d}}\right)\right|_{\text{c},\text{ d}=1} = 6\text{n}+30+56\text{n}+280+28\text{n}+140+80\text{n}+380$$
  $$\text{M}_{2}= 170\text{n}+830.$$
- Forgotten topological index (F)
  $$({\Delta}{\rm c}^{2} +{\Delta}{\rm d}^{2}) ({\mathcal{M}} (\text{G}))|_{\text{c},\text{ d}=1} =\text{ F}$$
  $$\left({\Delta}{\rm c}^{2} +{\Delta}{\rm d}^{2}\right)\left(\text{F}\left({\text{c,d}}\right)\right)= 5\left(3\text{n}+15\right)\text{c}^{1}\text{d}^{2}+20\left(7\text{n}+35\right)\text{c}^{2}\text{d}^{4}+17 \left(7\text{n}+35\right)\text{c}^{1}\text{d}^{4} +32\left(5\text{n}+25\right)\text{c}^{4}\text{d}^{4}$$
  $$({\Delta}{\rm c}^{2} +{\Delta}{\rm d}^{2})(\text{F}({\text{c,d}}))|_{\text{c},\text{ d}=1} = 15\text{n}+75+140\text{n}+700+119\text{n}+595+160\text{n}+800$$
  $$\text{F }= 434\text{n}+2170.$$
- Redefine third Zagreb index (ReZG_3_)
  $$({\Delta }_{\text{c}}{\Delta }_{\text{d}})({\Delta }_{\text{c}}+{\Delta }_{\text{d}})({\mathcal{M}} (\text{G}))|_{\text{c},\text{ d}=1} =\text{ ReZG}_{3}$$
  $$({\Delta }_{\text{c}}{\Delta }_{\text{d}})({\Delta }_{\text{c}}+{\Delta }_{\text{d}})({\mathcal{M}} (\text{G}))|\text{c},\text{ d}=1 =\text{ ReZG}_{3}$$
  $$\left({\Delta }_{\text{c}}{\Delta }_{\text{d}}\right)\left({\Delta }_{\text{c}}+{\Delta }_{\text{d}}\right)\left(\text{F}\left({\text{c,d}}\right)\right)= 6\left(3\text{n}+15\right)\text{c}^{1}\text{d}^{2}+48\left(7\text{n}+35\right)\text{c}^{2}\text{d}^{4}+ 20\left(7\text{n}+35\right)\text{c}^{1}\text{d}^{4} +128\left(5\text{n}+25\right)\text{c}^{4}\text{d}^{4}$$
  $$({\Delta }_{\text{c}}{\Delta }_{\text{d}}) ({\Delta }_{\text{c}}+{\Delta }_{\text{d}}) (\text{F}({\text{c,d}}))|_{{\text{c,d}}=1} =18\text{n}+90+336\text{n}+1680+140\text{n}+700+640\text{n}+3200.$$
  $$\text{ReZG}_{3} =1134\text{n}+5670.$$
- General Randić index (GR_α_)
  $$({\Delta }_{c}^{\alpha }{\Delta }_{d}^{\alpha }) ({\mathcal{M}} (\text{G}))|_{\text{c},\text{ d }=1} ={\rm GR} {\upalpha }$$
  $$\left({\Delta }_{{\varvec{c}}}^{\boldsymbol{\alpha }}{\Delta }_{{\varvec{d}}}^{\boldsymbol{\alpha }}\right)\left(\text{F }\left(\text{c},\text{ d}\right)\right)= 2{\upalpha}\left(3\text{n}+15\right)\text{c}^{1}\text{d}^{2} + 8{\upalpha }\left(7\text{n}+35\right)\text{c}^{2}\text{d}^{4} + 4{\upalpha}\left(7\text{n}+35\right)\text{c}^{1}\text{d}^{4} + 16{\upalpha}\left(5\text{n}+25\right)\text{c}^{4}\text{d}^{4}$$
  $$({\Delta }_{{\varvec{c}}}^{\boldsymbol{\alpha }}{\Delta }_{{\varvec{d}}}^{\boldsymbol{\alpha }})(\text{F}({\text{c,d}}))|_{\text{c},\text{ d}=1}=2{\upalpha}(3\text{n}+15) +8{\upalpha}(7\text{n}+35) + 4{\upalpha}(7\text{n}+35) + 16{\upalpha}(5\text{n}+25)$$
  $${\rm GR}{\upalpha}= 2{\upalpha}(3\text{n}+15) +8{\upalpha}(7\text{n}+35) +4{\upalpha}(7\text{n}+35) + 16{\upalpha}(5\text{n}+25).$$
- Modified second Zagreb index (^m^M_2_)
  $$({\text{I}}_{\rm{c}}{\text{I}}_{\rm{d}} ) ({\mathcal{M}} (\text{G}))|_{{\text{ c, d}}=1} =\text{ mM}_{2}$$
  $$\left({\text{I}}_{\rm{c}}{\text{I}}_{\rm{d}} \right)\left(\text{F}\left({\text{c,d}}\right)\right)= \frac{1}{2}\left(3\text{n}+15\right)\text{c}^{1}\text{d}^{2} +\frac{1}{8} \left(7\text{n}+35\right)\text{c}^{2}\text{d}^{4}+\frac{1}{4} \left(7\text{n}+35\right)\text{c}^{1}\text{d}^{4} + \frac{1}{16} \left(5\text{n}+25\right)\text{c}^{4}\text{d}^{4}$$
  $$({\text{I}}_{\rm{c}}{\text{I}}_{\rm{d}} ) (\text{F}({\text{c,d}}))|_{{\text{ c, d}}=1} = \frac{1}{2}(3\text{n}+15) +\frac{1}{8} (7\text{n}+35) +\frac{1}{4} (7\text{n}+35) + \frac{1}{16} (5\text{n}+25)$$
  $$\text{mM}_{2} = \frac{61}{16}\text{n}+\frac{355}{16}.$$
- Symmetric division deg index (SSD)
  $$({\Delta }_{\rm{c}}{\text{I}}_{\rm{d}} + {{\text{I}}_{\text{c}}\Delta }_{\text{d}}) ({\mathcal{M}} (\text{G}))|_{\text{c},\text{ d}=1} =\text{ SSD}$$
  $$\left({\Delta }_{\rm{c}}{\text{I}}_{\rm{d}} + {{\text{I}}_{\text{c}}\Delta }_{\text{d}}\right)\left(\text{F}\left({\text{c,d}}\right)\right)= \frac{1}{2}\left(3\text{n}+15\right)\text{c}^{1}\text{d}^{2} +\frac{2}{4} \left(7\text{n}+35\right)\text{c}^{2}\text{d}^{4}+\frac{1}{4} \left(7\text{n}+35\right)\text{c}^{1}\text{d}^{4} + \frac{4}{4} \left(5\text{n}+25\right)\text{c}^{4}\text{d}^{4}$$
  $$+ \frac{2}{1}\left(3\text{n}+15\right)\text{c}^{1}\text{d}^{2} +\frac{4}{2} \left(7\text{n}+35\right)\text{c}^{2}\text{d}^{4}+\frac{4}{1} \left(7\text{n}+35\right)\text{c}^{1}\text{d}^{4} + \frac{4}{4} \left(5\text{n}+25\right)\text{c}^{4}\text{d}^{4}$$
  $$({\Delta }_{\rm{c}}{\text{I}}_{\rm{d}} + {{\text{I}}_{\text{c}}\Delta }_{\text{d}})(\text{F}({\text{c,d}}))|_{\text{c},\text{ d}=1}= \frac{1}{2}(3\text{n}+15) +\frac{2}{4} (7\text{n}+35) + \frac{1}{4} (7\text{n}+35) + \frac{4}{4} (5\text{n}+25) +\frac{2}{1} \left(3\text{n}+15\right)$$
  $$+ \frac{4}{2} \left(7\text{n}+35\right)+ \frac{4}{1} \left(7\text{n}+35\right)+ \frac{4}{4} \left(5\text{n}+25\right)$$
  $$\text{SSD }= \frac{259}{4}\text{n}+\frac{1295}{4}.$$
- Harmonic index (H)
  $$2\text{Ic J }({\mathcal{M}} (\text{G})) |_{\text{c}=1}=\text{ H}$$
  $$2\text{Ic J }\left(\text{F}\left({\text{c,d}}\right)\right)= \frac{2}{3} \left(3\text{n}+15\right)\text{c}^{3}+\frac{2}{6} \left(7\text{n}+35\right)\text{c}^{6}+\frac{2}{5} \left(7\text{n}+35\right)\text{c}^{5} + \frac{2}{8} \left(5\text{n}+25\right)\text{c}^{8}$$
  $$2\text{IcJ }(\text{F}({\text{c,d}})) |_{\text{c}=1}= \frac{2}{3} (3\text{n}+15) +\frac{2}{6} (7\text{n}+35) +\frac{2}{5} (7\text{n}+35) + \frac{2}{8} (5\text{n}+25)$$
  $$\text{H }= \frac{503}{60}\text{n }+\frac{483}{12}.$$
- Inverse sum indeg index (ISI)
  $${\text{I}}_{\text{c}}\text{J}{\Delta }_{\text{c}}{\Delta }_{\text{d}} ({\mathcal{M}} (\text{G}))|_{\text{ c}=1} =\text{ ISI}$$
  $${\text{I}}_{\text{c}}\text{J}{\Delta }_{\text{c}}{\Delta }_{\text{d}} \left(\text{F}\left({\text{c,d}}\right)\right)= \frac{2}{3} \left(3\text{n}+15\right)\text{c}^{3}+ \frac{8}{6} \left(7\text{n}+35\right)\text{c}^{6} + \frac{4}{5} \left(7\text{n}+35\right)\text{c}^{5}+ \frac{16}{8} \left(5\text{n}+25\right)\text{c}^{8}$$
  $${\text{I}}_{\text{c}}\text{J}{\Delta }_{\text{c}}{\Delta }_{\text{d}} (\text{F}({\text{c,d}}))|_{\text{ c}=1}= \frac{2}{3} (3\text{n}+15) + \frac{8}{6} (7\text{n}+35) + \frac{4}{5} (7\text{n}+35) + \frac{16}{8} (5\text{n}+25)$$
  $$\text{ISI}= \frac{404}{15}\text{n}+\frac{404}{3}.$$
- Augmented Zagreb index (AZI)
  $${ I}_{c}^{3}\text{Q}-2{\rm J} {\Delta }^{3}{\rm c} {\Delta }^{3}\text{d }({\mathcal{M}} (\text{G}))|_{\text{ c}=1} =\text{ AZI}$$
  $${ I}_{c}^{3}\text{Q}-2\text{J}{\Delta }_{{\varvec{c}}}^{3}{\Delta }_{{\varvec{d}}}^{3} \left(\text{F}\left({\text{c,d}}\right)\right)= 8\left(3\text{n}+15\right)\text{c }+ 8 \left(7\text{n}+35\right)\text{c}^{4}+\frac{64}{27} \left(7\text{n}+35\right)\text{c}^{3} + \frac{512}{27}\left(5\text{n}+25\right)\text{c}^{6}.$$
  $${I}_{c}^{3}\text{Q}-2\text{J}{\Delta }_{{\varvec{c}}}^{3}{\Delta }_{{\varvec{d}}}^{3} (\text{F}({\text{c,d}}))|_{\text{ c}=1} = 8(3\text{n}+15) + 8 (7\text{n}+35) + \frac{64}{27} (7\text{n}+35) + \frac{512}{27} (5\text{n}+25)$$
  $$\text{AZI}= (\frac{5168}{27})\text{ n}+\frac{25840}{27}.$$
  $$\square$$

### Theorem 3

*Let P be the graph of cyclodextrin conjugate structure. Then, the* $${\mathcal{N}}$$$${\mathcal{M}}$$-polynomials is

$${\mathcal{N}}{\mathcal{M}} (\text{P}(\text{c},\text{ d})) = (3\text{c}^{2}\text{d}^{5}+2\text{c}4\text{d}^{8}+\text{c}4\text{d}^{9}+4\text{c}4\text{d}^{11}+\text{c}^{5}\text{d}^{8}+2\text{c}^{5}\text{d}^{11}+2\text{c}8\text{d}^{9}+3\text{c}8\text{d}^{11}+\text{c}9\text{d}^{11}+3\text{c}11\text{d}^{11})\text{ n}+ (15\text{c}^{2}\text{d}^{5}+10\text{c}4\text{d}^{8}+5\text{c}4\text{d}^{9}+20\text{c}4\text{d}^{11}+5\text{c}^{5}\text{d}^{8}+10\text{c}^{5}\text{d}^{11}+10\text{c}^{8}\text{d}^{4}+15\text{c}^{8}\text{d}^{11}+5\text{c}9\text{d}^{11}+15\text{c}11\text{d}^{11}).$$

### ***Proof***

Consider P as a cyclodextrin conjugate structural graph. Assume the set ${{\mathcal{N}}}^{p}$_(g, h)_ = {$rs$ ϵ E (P) |${\mathcal{N}}$(r) = g, ${\mathcal{N}}$(s) = h} consisting of all edges with Nbhd degree sum (s, t). We have edges partitions of Nbhd degree sum, that is,

$${{\mathcal{N}}}^{p}_{ (2, 5)} = \{ rs{ \epsilon } {\rm E} (\text{P}) |{\mathcal{N}}(\text{r}) =2,{\mathcal{N}}(\text{s}) =5\},$$

$${{\mathcal{N}}}^{p} _{(4, 8)} = \{ { rs} \epsilon {\rm E} (\text{P}) |{\mathcal{N}}(\text{r}) =4,{\mathcal{N}}(\text{s}) =8\},$$

$${{\mathcal{N}}}^{p} _{(4, 9)} = \{ { rs} \epsilon {\rm E} (\text{P})|{\mathcal{N}}(\text{r}) =4,{\mathcal{N}}(\text{s}) =9\},$$

$${{\mathcal{N}}}^{p}_{ (4, 11)} = \{ { rs} \epsilon {\rm E} (\text{P})|{\mathcal{N}}(\text{r}) =4,{\mathcal{N}}(\text{s}) =11\},$$

$${{\mathcal{N}}}^{p}_ {(5, 8)} = \{ { rs} \epsilon {\rm E} (\text{P}) |{\mathcal{N}}(\text{r}) =5,{\mathcal{N}}(\text{s}) =8\},$$

$${{\mathcal{N}}}^{p} _{(5, 11)} = \{ { rs} \epsilon {\rm E} (\text{P}) |{\mathcal{N}}(\text{r}) =5,{\mathcal{N}}(\text{s}) =11\},$$

$${{\mathcal{N}}}^{p}_ {(8, 9)} = \{ { rs} \epsilon {\rm E} (\text{P}) |{\mathcal{N}}(\text{r}) =8,{\mathcal{N}}(\text{s}) =9\},$$

$${{\mathcal{N}}}^{p} _{(8, 11)} = \{ { rs} \epsilon {\rm E} (\text{P}) |{\mathcal{N}}(\text{r}) =8,{\mathcal{N}}(\text{s}) =11\},$$

$${{\mathcal{N}}}^{p}_ {(9, 11)} = \{ { rs} \epsilon {\rm E} (\text{P}) |{\mathcal{N}}(\text{r}) =9,{\mathcal{N}}(\text{s}) =11\},$$

$${{\mathcal{N}}}^{p}_ {(11, 11)} = \{ { rs} \epsilon {\rm E} (\text{P}) |{\mathcal{N}}(\text{r}) =11,{\mathcal{N}}(\text{s}) =11\}.$$

Then we have,

$$| {{\mathcal{N}}}^{p}_{(2, 5)}|=15+3\text{n}, |{{\mathcal{N}}}^{p}_{(4, 8)}|=10+2\text{n}, | {{\mathcal{N}}}^{p}_{(4, 9)}|=5+\text{n}, | {{\mathcal{N}}}^{p}_{(4, 11)}| =20+4\text{n}, | {{\mathcal{N}}}^{p}_{(5, 8)}| =5+\text{n}, | {{\mathcal{N}}}^{p}_{(5, 11)}| =10+2\text{n}, | {{\mathcal{N}}}^{p}_{(8, 9)}| =10+2\text{n}, | {{\mathcal{N}}}^{p}_{(8, 11)}| =15+3\text{n}, | {{\mathcal{N}}}^{p}_{(9, 11)}| =5+\text{n}, | {{\mathcal{N}}}^{p}_{(11, 11)} |=15+3\text{n},$$

By the definition of $${\mathcal{N}}$$$${\mathcal{M}}$$-polynomial, we have

$${\mathcal{N}}{\mathcal{M}}(L)=\sum_{g\le h}|{{{\mathcal{N}}}^{p}}_{(g, h)}|{c}^{g}{d}^{h}$$

$${\mathcal{N}}{\mathcal{M}}(L) =| {{\mathcal{N}}}^{p}_{(2, 5)}|\text{ c}^{2}\text{d}^{5}+ | {{\mathcal{N}}}^{p}_{(4, 8)}|\text{ c}^{4} \text{d}^{8}+| {{\mathcal{N}}}^{p}_{(4, 9)}|\text{ c}^{4} \text{d}^{9}+| {{\mathcal{N}}}^{p}_{(4, 11)}|\text{ c}^{4}\text{d}^{11} +| {{\mathcal{N}}}^{p}_{(5, 8)}|\text{c}^{5}\text{d}^{8} + | {{\mathcal{N}}}^{p}_{(5, 11)}| \times \text{c}^{5}\text{d}^{11} +| {{\mathcal{N}}}^{p}_{(8, 9)}|\text{c}^{8}\text{d}^{9}+| {{\mathcal{N}}}^{p}_{(8, 11)}|\text{c}^{8}\text{d}^{11} +| {{\mathcal{N}}}^{p}_{(9, 11)}|\text{c}^{9}\text{d}^{11} + | {{\mathcal{N}}}^{p}_{(11, 11)}|\text{c}^{11}\text{d}^{11}.$$

Putting all the values we get,

$${\mathcal{N}}{\mathcal{M}} (\text{P}(\text{c},\text{ d})) = (15+3\text{n})\text{ c}^{2} \text{d}^{5} + (10+2\text{n})\text{ c}^{4} \text{d}^{8} + (5+\text{n})\text{ c}^{4} \text{d}^{9}+ (20+4\text{n})\text{ c}^{4}\text{d}^{11} + (5+\text{n})\text{c}^{5}\text{d}^{8} + (10+2\text{n})\text{c}^{5}\text{d}^{11} + (10+2\text{n})\text{c}^{8}\text{d}^{9}+ (15+3\text{n})\text{c}^{8}\text{d}^{11}+ (5+\text{n})\text{c}^{9}\text{d}^{11} + (15+3\text{n})\text{c}^{11}\text{d}^{11}.$$

So,

$${\mathcal{N}}{\mathcal{M}} (\text{P}(\text{c},\text{ d})) = (3\text{c}^{2}\text{d}^{5}+2\text{c}4\text{d}^{8}+\text{c}4\text{d}^{9}+4\text{c}4\text{d}^{11}+\text{c}^{5}\text{d}^{8}+2\text{c}^{5}\text{d}^{11}+2\text{c}^{8}\text{d}^{9}+3\text{c}^{8}\text{d}^{11}+\text{c}9\text{d}^{11}+3\text{c}^{11}\text{d}^{11})\text{ n }+ (15\text{c}^{2}\text{d}^{5}+10\text{c}4\text{d}^{8}+5\text{c}4\text{d}^{9}+20\text{c}^{4}\text{d}^{11}+5\text{c}^{5}\text{d}^{8}+10\text{c}^{5}\text{d}^{11}+10\text{c}^{8}\text{d}^{4}+15\text{c}^{8}\text{d}^{11}+5\text{c}^{9}\text{d}^{11}+15\text{c}^{11}\text{d}^{11}).$$

$$\square$$

### Theorem 4

*If P represents a molecular structure of the conjugate cyclodextrins, then the *${\mathcal{N}}$ ${\mathcal{M}}$-*Polynomials indices are*

- *Nbhd first Zagreb index* (^n^${\mathcal{M}}$_1_) = 340n + 1700,
- *Nbhd second Zagreb index* (^n^${\mathcal{M}}$_2_) = 1326n + 6630,
- *Nbhd forgotten topological index* (^n^F) = 3046n + 15,230,
- *Nbhd redefine third Zagreb index* (^n^ReZG_3_) = 23796n + 118,980,
- *Nbhd general Randić index* (^n^GR_α_) = 10^α^ (15 + 3n) + 32^α^ (10 + 2n) + 36^α^ (5 + n) + 44^α^ (20 + 4n) + 40^α^ (5 + n) + 55^α^ (10 + 2n) + 72^α^ (72 + 2n) + 88^α^ (15 + 3n) + 99^α^ (5 + n) + 121^α^ (15 + 3n),
- *Nbhd modified second Zagreb index* (^nm^${\mathcal{M}}$_2_) = 0.37637n + 3.1965,
- *Nbhd symmetric division deg index* (^n^SSD) = 54.758n + 273.79,
- *Nbhd harmonic index* (^n^H) = 3.3666n + 16.833,
- *Nbhd inverse sum indeg index* (^n^ISI) = 88.145n + 440.72,
- *Nbhd augmented Zagreb index* (^n^AZI) = 1917.047n + 9585.238.

### Proof

Suppose P is a molecular graph of cyclodextrins and P(c,d) is the polynomial defined in Theorem 3. By following the structure of Theorem 2, one can find the required result. For a detailed proof, please see the Supplementary File.$\square$

## Linear regression model

In this section, we proposed the QSPR among the topological indices and some physicochemical characteristics of the antiviral drugs named; Lopinavir, Thalidomide, Hydroxychloroquine, Arbidol, Theaflavin, Ritonavir, Chloroquine and Remdesivir. We considered all degree-based and neighborhood degree sum-based topological indices to model nine physicochemical properties, namely, surface tension (T), enthalpy of vaporization (EoV), flash point (FP), polarizability (P), polar surface area (PSA), mass (M), molar volume (MV), molar refractivity (MR) and boiling point (BP). The drug structures and physicochemical characteristics are taken from https://www.chemspider.com/. As we know computation indices for unit structure is very easy. We considered all indices based on polynomials. Here, we are adding the calculation of only significant models whose value of the correlation coefficient is greater than 0.850. The statistical analysis was performed using software IBM SPSS Statistics version 26.0 available at https://www.ibm.com/support/pages/downloading-ibm-spss-statistics-26. Table 4 shows the correlation between physical properties and M-polynomial indices, and Table 5 shows the correlation between physical properties and $${\mathcal{N}}$$$${\mathcal{M}}$$—polynomials indices.

**Table 4:** Linear regression model between physical properties and M-polynomial indices.

Model	R	R^2^	RMSE	Adj(R^2^)
BP = 93.33 + 3.098M_2_	0.9921	0.9844	33.13	0.9812
FP = 32.6 + 43.17^m^M_2_	0.956	0.914	39.43	0.8968
P = − 1.199 + 6.973^m^M_2_	0.9801	0.9606	3.85	0.955
PSA = − 36.61 + 0.1621ReZG_3_	0.8631	0.745	38.83	0.7086
MV = − 27.18 + 51.43^m^M_2_	0.924	0.8538	58.01	0.8329
MR = − 3.023 + 17.59^m^M_2_	0.9799	0.9603	9.747	0.9547
EoV = 20.42 + 0.4502M_2_	0.9888	0.9779	5.739	0.9735
M = 4.383 + 64.23^m^M_2_	0.9879	0.976	27.44	0.9726

**Table 5:** Linear regression model between physical properties and $${\mathcal{N}}$$$${\mathcal{M}}$$-polynomials indices.

Model	R	R^2^	RMSE	Adj(R^2^)
BP = 95.59 + 6.324^n^ISI	0.9921	0.9844	33.08	0.9813
BP = 94.44 + 3.131 ^n^${\mathcal{M}}$_2_	0.9921	0.9844	33.06	0.9813
BP = 93.33 + 1.549 ^n^${\mathcal{M}}$_1_	0.9921	0.9844	33.13	0.9812
EoV = 20.58 + 0.4549 ^n^H	0.9888	0.9779	5.742	0.9735
EoV = 20.42 + 0.2251 ^n^${\mathcal{M}}$_1_	0.9888	0.9779	5.739	0.9735
PSA = − 34.22 + 0.06719 ^n^F	0.863	0.7448	38.85	0.7083
FP = 38.33 + 46.41 ^n^H	0.9637	0.9288	35.88	0.9146
MV = − 54.69 + 293.5 ^nm^${\mathcal{M}}$_2_	0.9825	0.9655	28.18	0.9606
MR = − 5.824 + 95.56 ^nm^${\mathcal{M}}$_2_	0.9918	0.9838	6.221	0.9815
M = 18 + 67.67 ^n^${\mathcal{M}}$_2_	0.9842	0.9687	31.38	0.9642
P = − 2.303 + 37.88 ^nm^${\mathcal{M}}$_2_	0.9919	0.9839	2.461	0.9816

The examined M-polynomial and $${\mathcal{N}}$$$${\mathcal{M}}$$-polynomial indices are strongly correlated with the physicochemical properties of the drugs.

(i) The ^n^${\mathcal{M}}$_1_, M_2_, ^n^${\mathcal{M}}$_2_ and ^n^ISI for the BP with correlation coefficient R = 0.9921.
(ii) The ^n^H for the FP with correlation coefficient R = 0.9637
(iii) The ^nm^${\mathcal{M}}$_2_ for the MV with correlation coefficient R = 0.9825.
(iv) The ^nm^${\mathcal{M}}$_2_ for the MR with correlation coefficient R = 0.9918.
(v) The M_2_, ^n^${\mathcal{M}}$_1_ and ^n^H for the EoV with correlation coefficient R = 0.9888.
(vi) The ^nm^${\mathcal{M}}$_2_ for the P with correlation coefficient R = 0.9919.
(vii) The ReZG_3_ and ^n^F for the PSA with correlation coefficient R = 0.8631.
(viii) The ^n^${\mathcal{M}}$_2_ for the mass (M) with correlation coefficient R = 0.9879.

Linear regression was preferred due to its simplicity, ease of interpretation, efficiency, and suitability for initial exploration and analysis of the relationship between topological indices and physicochemical properties. While it has its limitations (like sensitivity to outliers, multicollinearity and underfitting), its advantages often make it the first choice for predictive modeling in many scientific studies.

## Numerical results and discussion

Applying topological indices that are based on the $${\mathcal{M}}$$-polynomial and neighborhood $${\mathcal{N}}$$$${\mathcal{M}}$$-polynomial, various dissimilar physicochemical features can still be forecasted with an elevated level of reliability. Using the $${\mathcal{M}}$$-polynomial and ˔$${\mathcal{N}}$$$${\mathcal{M}}$$-polynomial strategies, Kirmani et al.^41^ examined several medicines that are antiviral for COVID-19. Kirmani et al. found that to predict the boiling point and temperature of condensation one can apply the F-index, neighborhood inverse sum and first Zagreb index, to determine flash points we can use the neighborhood Randić and modified second Zagreb indices, and to predict molar refractivity, polarity and molar volume we can use the Randić and neighborhood modified second Zagreb and redefined third Zagreb index, respectively.

Topological indices can be used to analyze the physical and chemical properties of pharmaceuticals, as Kirmani proved in the QSPR study. The association between polarization, molar refractivity and flashpoints is demonstrated by the neighborhood degree sum derived from the modified second Zagreb index. Also, we examined the relationship between indices and physicochemical characteristics of antiviral drugs and showed that the computed indices predict the physicochemical characteristics.

We can claim that for the previously stated reasons and based on the results given in “Linear regression model”, the topological indices deliberated overhead are valuable instruments for the QSPR study of antiviral drugs. QSPR models can dramatically reduce the expenses of time, workers and physical resources. To understand the biological behavior of antiviral medications that are extensively studied, the power source QSPR/QSAR model-based descriptive approach is specifically applied.

**Figure 2:**
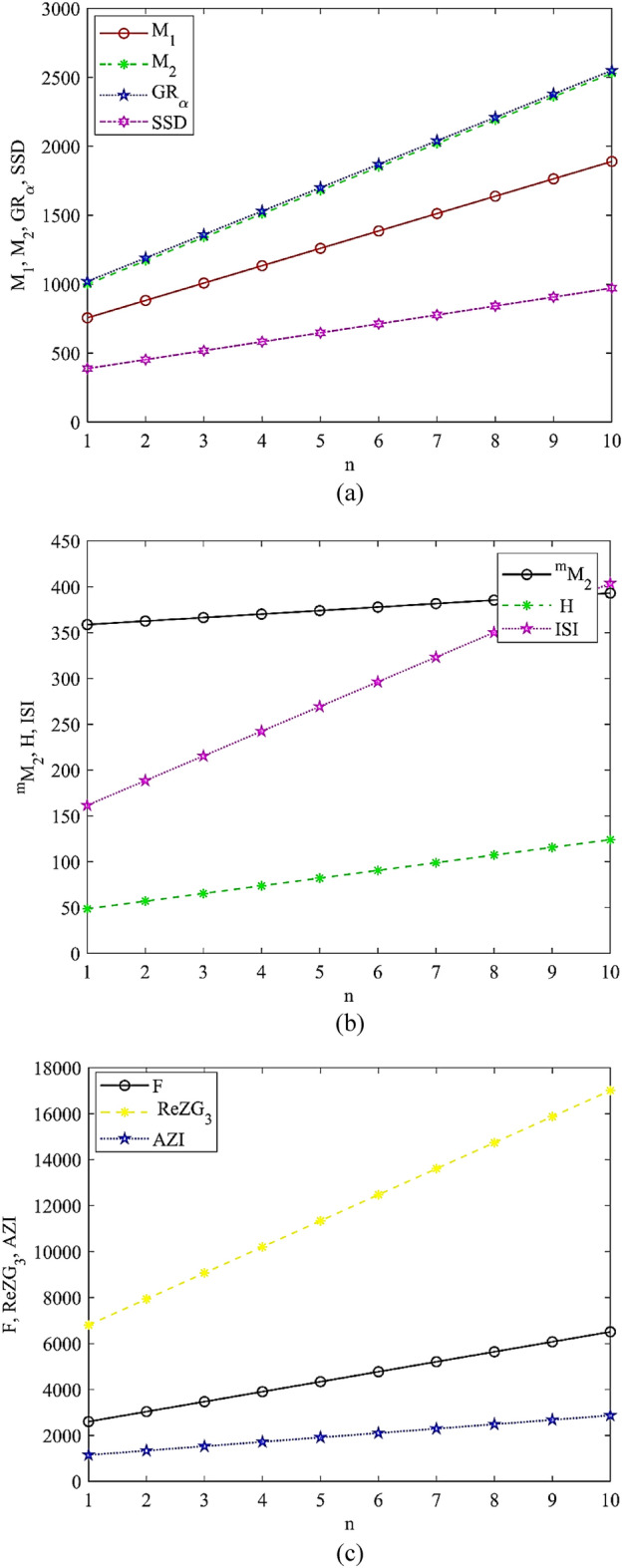
Graphical visualization of M-polynomial indices: **(a)** first Zagreb, second Zagreb, general Randić and symmetric division deg index, **(b)** modified second Zagreb, harmonic and inverse sum indeg index, and **(c)** forgotten, redefine third Zagreb and augmented Zagreb index.

**Figure 3:**
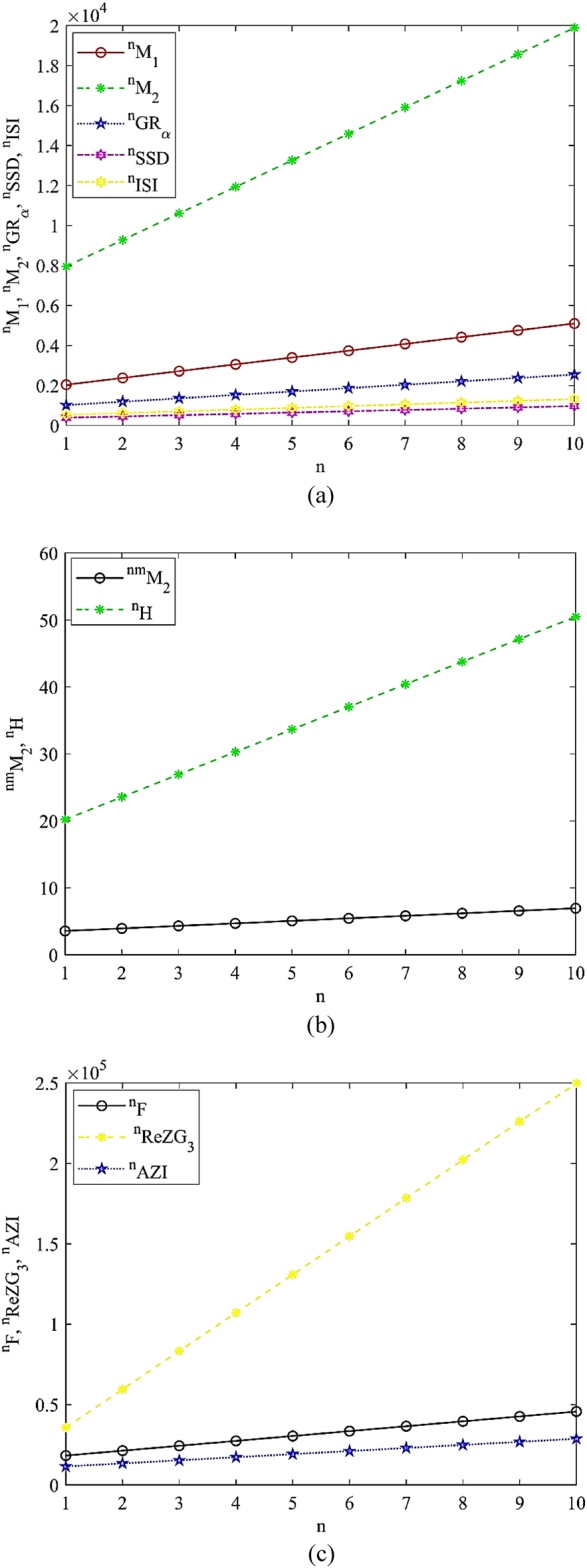
Graphical visualization of Nbhd M-polynomial indices: **(a)** Nbhd first Zagreb, Nbhd second Zagreb, Nbhd general Randić and Nbhd symmetric division deg and Nbhd inverse sum indeg index, **(b)** Nbhd modified second Zagreb and Nbhd harmonic index, and **(c)** Nbhd forgotten, Nbhd redefine third Zagreb and Nbhd augmented Zagreb index.

We have provided numerical results describing degree-based as well as neighborhood sum-based topological indices for the chemical structure of cyclodextrins conjugate under this segment using the $${\mathcal{M}}$$-polynomial and $${\mathcal{N}}$$$${\mathcal{M}}$$-polynomial methods. By using the already proposed models calculated through QSPR study for $${\mathcal{M}}$$-polynomial and $${\mathcal{N}}$$$${\mathcal{M}}$$-polynomial and our computed numerical results for cyclodextrins conjugate one can easily calculate the physicochemical characteristics of boiling point, temperature of condensation, flash point, molar refractivity, polarity and molar volume. Additionally, we plotted the graphical visualization of calculated numerical results which is depicted in Figs. 2 and 3.

## Conclusion

The utilization of topological indices in quantitative structure–property relationships has significantly broadened the scope of drug discovery and development, particularly in the field of anti-parasitic and antiviral medications. In this study, we computed the degree-based M-polynomial and neighbor degree-based M-polynomial indices, along with graphical representations of cyclodextrin structure, in elucidating the pharmacological characteristics of the compound. By calculating various topological indices derived from these polynomials, we provided valuable insights into the physicochemical properties of antiviral treatments, facilitating predictive modeling and property forecasting. We examined the physicochemical characteristics of antiviral drugs that can be predicted by these topological indices. Furthermore, our findings underscore the importance of topological indices in guiding the design and optimization of drug structures, with implications extending to the development of chemotherapeutic agents for cancer treatment.

### Supplementary Information

Supplementary Information.

## Data Availability

All data generated or analysed during this study are included in this published article.
