# Supplementary material for: Exploring physicochemical characteristics of cyclodextrin through M-polynomial indices
Source: Sci Rep. 2024 Aug 28;14:20029. doi: 10.1038/s41598-024-68775-z (PMC11358294; doi:10.1038/s41598-024-68775-z)
Supplement: Supplementary file 1 — Supplementary Information. [file 41598_2024_68775_MOESM1_ESM.docx]

Exploring Physicochemical Characteristics of Cyclodextrin Through M-Polynomial Indices

Abdul Rauf^1^, Muhammad Naeem^2^, Rahila Ramzan^1^, Alhagie Cham^3*^

2

Department of Mathematics, Air University Multan Campus, Multan, Pakistan

attari ab092@yahoo.com

^1^Department of Mathematics, Air University Multan Campus, Multan, Pakistan

attari_[ab092@yahoo.com](mailto:ab092@yahoo.com); [mirzasaim049@gmail.com](mailto:mirzasaim049@gmail.com);

Department of Mathematics, School of Natural Sciences,

^2^National University of Sciences and Technology (NUST), Islamabad, Pakistan

[naeemjutt5530@gmail.com](mailto:naeemjutt5530@gmail.com);

^3^ School of Arts and Sciences, University of The Gambia, Banjul, The Gambia [alhagie.cham@utg.edu.gm](mailto:alhagie.cham@utg.edu.gm);

*Corresponding author: Alhagie Cham, alhagie.cham@utg.edu.gm

***Proof of Theorem 4***. The neighbourhood (Nbhd) degrees accumulation based on the Ɲϻ-polynomial of the P structure is valuable.

P(c, d) = (15+3n ) c^2^d^5^ + (10+2n)c^4^d^8^ + (5+n) c^4^d^9^ + (20+4n) c^4^d^11^ + (5+n)c^5^d^8^ + (10+2n) c^5^d^11^  + (10+2n) c^8^d^9^ + (15+3n) c^8^d^11^ + (5+n) c^9^d^11^ + (15+3n) c^11^d^11^ .

Then,

**a. Nbhd first Zagreb index (^n^ϻ_1_)**

($\Delta_{c}$+$\Delta_{d}$) (P(c, d)) |_c, d=1_ = ^n^ϻ_1_

($\Delta_{c}$+$\Delta_{d}$)P(c, d) =7(15+3n) c^2^d^5^+12(10+2n) c^4^d^8^+13(5+n) c^4^d^9^ +15(20+4n) c^4^d^11^+13(5+n) c^5^d^8^

+ 16(10+2n) c^5^d^11^+17(10+2n) c^8^d^9^ +19(15+3n) c^8^d^11^+20 (5+n) c^9^d^11^

+22 (15+3n) c^11^ d^11^

($\Delta_{c}$+$\Delta_{d}$) (P(c, d))|_c, d=1_=7(15+3n) +12(10+2n) +13(5+n) +15(20+4n) +13(5+n) + 16(10+2n) +17(10+2n) +19(15+3n) +20 (5+n) +22(15+3n)

^n^ϻ_1_ =340n+1700.

**b. Nbhd second Zagreb index (^n^ϻ_2_)**

($\Delta_{c}\Delta_{d}$) (P(c, d))| _c, d=1_ = ^n^ϻ_2_

($\Delta_{c}\Delta_{d}$) P(c, d) = 10(15+3n) c^2^d^5^ + 32(10+2n) c^4^d^8^ + 36(5+n) c^4^d^9^ + 44(20+4n) c^4^d^11^

+ 40(5+n) c^5^d^8^ + 55(10+2n) c^5^d^11^ + 72(10+2n) c^8^d^9^ + 88(15+3n) c^8^d^11^

+ 99(5+n) c^9^d^11^ + 121(15+3n) c^11^ d^11^

($\Delta_{c}\Delta_{d}$) (P(c, d))| _c, d=1_= 10(15+3n) + 32(10+2n) + 36(5+n) + 44(20+4n) + 40(5+n) + 55(10+2n)

+ 72(10+2n) + 88(15+3n) + 99(5+n) + 121 (15+3n)

^n^ϻ_2_ = 1326n+6630.

**c. Nbhd forgotten index (^n^F)**

($\boldsymbol{\Delta}_{\boldsymbol{c}}^{\boldsymbol{2}}\boldsymbol{+}\boldsymbol{\Delta}_{\boldsymbol{d}}^{\boldsymbol{2}}$) (P(c, d))| _c, d =1_= ^n^F

($\boldsymbol{\Delta}_{\boldsymbol{c}}^{\boldsymbol{2}}\boldsymbol{+}\boldsymbol{\Delta}_{\boldsymbol{d}}^{\boldsymbol{2}}$) (P(c, d)) = 29(15+3n) c^2^ d^5^ + 80(10+2n) c^4^d^8^ + 97(5+n) c^4^d^9^ + 137(20+4n) c^4^d^11^

+ 89(5+n) c^5^ d^8^ + 146(10+2n) c^5^d^11^ + 145(10+2n) c^8^ d^9^ + 185(15+3n) c^8^d^11^

+ 202(5+n) c^9^d^11^ + 242 (15+3n) c^11^ d^11^

($\boldsymbol{\Delta}_{\boldsymbol{c}}^{\boldsymbol{2}}\boldsymbol{+}\boldsymbol{\Delta}_{\boldsymbol{d}}^{\boldsymbol{2}}$) (P(c, d))| _c, d =1_= 29 (15+3n) + 80(10+2n) + 97(5+n) + 137(20+4n) + 89(5+n)

+ 146(10+2n) + 145(10+2n) + 185(15+3n) + 202(5+n) + 242 (15+3n)

^n^F = 3046n+15230.

**d. Nbhd Redefine third Zagreb index (^n^ReZG_3_)**

($\Delta_{c}\Delta_{d}$) ($\Delta_{c}{+\Delta}_{d}$) (P(c, d))| _c, d=1_= ^n^ReZG_3_

($\Delta_{c}\Delta_{d}$) ($\Delta_{c}{+\Delta}_{d}$) (P(c, d)) = (15+3n) c^2^ d^5^+384(10+2n) c^4^d^8^ + 468(5+n) c^4^d^9^ + 660(20+4n) c^4^d^11^  + 520(5+n) c^5^ d^8^ + 880(10+2n) c^5^d^11^ +1224(10+2n) c^8^ d^9^ +1672(15+3n) × c^8^d^11^ + 1980(5+n) c^9^d^11^+2662 (15+3n) c^11^ d^11^

^n^ReZG_3_ = 23796n+118980.

**e. Nbhd General Randić index (^n^GR_α_)**

$\boldsymbol{(}\boldsymbol{\Delta}_{\boldsymbol{c}}^{\boldsymbol{\alpha}}\boldsymbol{\Delta}_{\boldsymbol{d}}^{\boldsymbol{\alpha}}\boldsymbol{)}$ (P(c, d))|_c, d= 1_ = ^n^GRα

$\boldsymbol{(\Delta}_{\boldsymbol{c}}^{\boldsymbol{\alpha}}\boldsymbol{\Delta}_{\boldsymbol{d}}^{\boldsymbol{\alpha}}\boldsymbol{)}$ (P(c, d))= 10^α^ (15 +3n) c^2^ d^5^ + 32^α^ (10+2n) c^4^d^8^

+ 36^α^ (5+n) c^4^d^9^ + 44^α^ (20+4n) c^4^d^11^ + 40^α^ (5+n) c^5^d^8^ + 55 ^α^ (10+2n) c^5^d^11^

+ 72^α^ (10+2n) c^8^d^9^ + 88^α^ (15+3n) c^8^d^11^ + 99^α^ (5+n) c^9^ d^11^ + 121^α^ (15+3n) c^11^ d^11^

$\boldsymbol{(\Delta}_{\boldsymbol{c}}^{\boldsymbol{\alpha}}\boldsymbol{\Delta}_{\boldsymbol{d}}^{\boldsymbol{\alpha}}\boldsymbol{)}$ (P(c, d))|_c, d=1_= 10^α^ (15 +3n) + 32^α^ (10+2n) + 36^α^ (5+n) + 44^α^ (20+4n) + 40^α^ (5+n)

+ 55^α^ (10+2n) + 72 ^α^ (10+2n) + 88^α^ (15+3n) + 99^α^ (5+n) + 121^α^ (15+3n)

**f. Nbhd Modified second Zagreb index (^nm^ϻ_2_)**

($I_{c}I_{d}$ ) (P(c, d))| _c, d=1_ = ^nm^ϻ_2_

($I_{c}I_{d}$ ) (P(c, d)) =$\frac{1}{10}$ (15 +3n) c^2^d^5^+$\frac{1}{32}$ (10+2n) c^4^d^8^+$\frac{1}{36}$ (5+n) c^4^d^9^ +$\frac{1}{44}$ (20+4n) c^4^ d^11^

+$\frac{1}{40}$ (5+n) c^5^d^8^+$\frac{1}{55}$ (10+2n) c^5^d^11^+$\frac{1}{72}$ (10+2n) c^8^d^9^ +$\frac{1}{88}$ (15+3n) c^8^d^11^

+$\frac{1}{99}$ (5+n) c^9^d^11^+$\frac{1}{121}$ (15+3n) c^11^ d^11^

($I_{c}I_{d}$ ) (P(c, d))| _c, d=1_= $\frac{1}{10}$ (15 +3n) +$\frac{1}{32}$ (10+2n) +$\frac{1}{36}$ (5+n) +$\frac{1}{44}$ (20+4n) +$\frac{1}{40}$ (5+n) +$\frac{1}{55}$ (10+2n)

+$\frac{1}{72}$ (10+2n) +$\frac{1}{88}$ (15+3n) +$\frac{1}{99}$ (5+n) +$\frac{1}{121}$ (15+3n)

^nm^ϻ_2_ = 0.37637n+3.1965

**g. Nbhd Symmetric division deg index (^n^SSD)**

($\Delta_{c}I_{d}$ + ${I_{c}\Delta}_{d}$) (P(c, d))| _c, d=1_= ^n^SSD

($\Delta_{c}I_{d}$ + ${I_{c}\Delta}_{d}$) (P(c, d))= $\frac{2}{5}$ (15 +3n) c^2^d^5^ +$\frac{4}{8}$ (10+2n) c^4^d^8^ +$\frac{4}{9}$ (5+n) c^4^d^9^ +$\frac{4}{11}$ (20+4n) c^4^ d^11^

+$\frac{5}{8}$ (5+n) c^5^d^8^ +$\frac{5}{11}$ (10+2n) c^5^d^11^ +$\frac{8}{9}$ (10+2n) c^8^d^9^ +$\frac{8}{11}$ (15+3n) c^8^d^11^

+$\frac{9}{11}$ (5+n) c^9^d^11^ +$\frac{11}{11}$ (15+3n) c^11^ d^11^ +$\frac{5}{2}$ (15 +3n) c^2^d^5^ +$\frac{8}{4}$ (10+2n) c^4^d^8^

+$\frac{9}{4}$ (5+n) c^4^d^9^+$\frac{11}{4}$ (20+4n) c^4^ d^11^ +$\frac{8}{5}$ (5+n) c^5^d^8^ +$\frac{11}{5}$ (10+2n) c^5^d^11^

+$\frac{9}{8}$ (10+2n) c^8^d^9^ +$\frac{11}{8}$ (15+3n) c^8^d^11^ + $\frac{11}{9}$ (5+n) c^9^d^11^+$\frac{11}{11}$ (15+3n) c^11^ d^11^

($\Delta_{c}I_{d}$ + ${I_{c}\Delta}_{d}$) (P(c, d))|_c, d=1_ = $\frac{2}{5}$ (15 +3n) + $\frac{4}{8}$ (10+2n) +$\frac{4}{9}$ (5+n) +$\frac{4}{11}$ (20+4n) +$\frac{5}{8}$ (5+n)

+$\frac{5}{11}$ (10+2n) + $\frac{8}{9}$ (10+2n) +$\frac{8}{11}$ (15+3n) +$\frac{9}{11}$ (5+n) +$\frac{11}{11}$ (15+3n)

+$\frac{5}{2}$ (15 +3n) +$\frac{8}{4}$ (10+2n) +$\frac{9}{4}$ (5+n) +$\frac{11}{4}$ (20+4n) +$\frac{8}{5}$ (5+n) +$\frac{11}{5}$ (10+2n)

+$\frac{9}{8}$ (10+2n) +$\frac{11}{8}$ (15+3n) +$\frac{11}{9}$ (5+n) +$\frac{11}{11}$ (15+3n)

^n^SSD =54.758n+273.79

**h. Nbhd Harmonic index (^n^H)**

2I_c_J (P(c, d))| _c=1_= ^n^H

2I_c_J (P(c, d)) =$\frac{2}{7}$ (15 +3n) c^7^+$\frac{1}{6}$ (10+2n) c^12^+$\frac{2}{13}$ (5+n) c^13^+$\frac{1}{22}$ (20+4n) c^15^  +$\frac{2}{3}$ (5+n) c^13^

+$\frac{1}{8}$ (10+2n) c^16^+$\frac{2}{17}$ (10+2n) c^1^+$\frac{2}{19}$ (15+3n) c^19^+$\frac{1}{10}$ (5+n) c^20^+$\frac{1}{11}$ (15+3n) c^22^

2I_c_J (P(c, d))| _c=1_ = $\frac{2}{7}$ (15 +3n) +$\frac{1}{6}$ (10+2n) + $\frac{2}{13}$ (5+n) +$\frac{1}{22}$ (20+4n) +$\frac{2}{3}$ (5+n) +$\frac{1}{8}$ (10+2n)

+ $\frac{2}{17}$ (10+2n) +$\frac{2}{19}$ (15+3n) + $\frac{1}{10}$ (5+n) +$\frac{1}{11}$ (15+3n)

^n^H =3.3666n+16.833

**i. Nbhd Inverse sum indeg index (^n^ISI)**

$I_{c}$J$\Delta_{c}\Delta_{d}$ (P(c, d))| _c=1_ = ^n^ISI

$I_{c}$J$\Delta_{c}\Delta_{d}$ (P(c, d)) =$\frac{10}{7}$ (15 +3n) c^2^d^5^ + $\frac{32}{12}$ (10+2n) c^4^d^8^ + $\frac{36}{13}$ (5+n) c^4^d^9^ + $\frac{44}{15}$ (20+4n) c^4^ d^11^

+$\frac{40}{13}$ (5+n) c^5^d^8^+$\frac{55}{16}$ (10+2n) c^5^d^11^+$\frac{72}{17}$ (10+2n) c^8^d^9^ + $\frac{88}{19}$ (15+3n) c^8^d^11^

+$\frac{99}{20}$ (5+n) c^9^d^11^+$\frac{121}{22}$ (15+3n) c^11^ d^11^

$I_{c}$J$\Delta_{c}\Delta_{d}$ (P(c, d))| _c=1_= $\frac{10}{7}$ (15 +3n) +$\frac{32}{12}$ (10+2n) + $\frac{36}{13}$ (5+n) +$\frac{44}{15}$ (20+4n) +$\frac{40}{13}$ (5+n)

+ $\frac{55}{16}$ (10+2n) +$\frac{72}{17}$ (10+2n) +$\frac{88}{19}$ (15+3n) +$\frac{99}{20}$ (5+n) +$\frac{121}{22}$ (15+3n)

^n^IS = 88.145n+440.72

**j. Nbhd Augmented Zagreb index (^n^AZI)**

I^3^_c_Q_-2_JΔ^3^_c_Δ^3^_d_ (P(c, d))| _c=1_ = ^n^AZI

$I_{c}^{3}$Q_-2_J$\boldsymbol{\Delta}_{\boldsymbol{c}}^{\boldsymbol{3}}\boldsymbol{\Delta}_{\boldsymbol{d}}^{\boldsymbol{3}}$ (P(c, d)) = 8 (15 +3n) c^5^+$\frac{4096}{125}$ (10+2n) c^10^ +$\frac{46656}{1331}$ (5+n) c^11^ +$\frac{85184}{2197}$ (20+4n) c^13^ $+ \frac{64000}{1331}$ (5+n) c^11^ +$\frac{166375}{2744}$ (10+2n) c^14^ +$\frac{373248}{3375}$ (10+2n) c^15^

+$\frac{681472}{4913}$ (15+3n) c^17^+$\frac{970299}{5832}$ (5+n) c^18^ +$\frac{1771561}{8000}$ (15+3n) c^20^

$I_{c}^{3}$Q_-2_J$\boldsymbol{\Delta}_{\boldsymbol{c}}^{\boldsymbol{3}}\boldsymbol{\Delta}_{\boldsymbol{d}}^{\boldsymbol{3}}$ (P(c, d))| _c=1_ **=** 8 (15 +3n) +$\frac{4096}{125}$ (10+2n) +$\frac{46656}{1331}$ (5+n) +$\frac{85184}{2197}$ (20+4n)

+$\frac{64000}{1331}$ (5+n) +$\frac{166375}{2744}$ (10+2n) +$\frac{373248}{3375}$ (10+2n) +$\frac{681472}{4913}$ (15+3n)

+$\frac{970299}{5832}$ (5+n) +$\frac{1771561}{8000}$ (15+3n)

^n^AZI =1917.047n +9585.238.
